# Detection and Tracking of Dynamic Objects by Using a Multirobot System: Application to Critical Infrastructures Surveillance

**DOI:** 10.3390/s140202911

**Published:** 2014-02-12

**Authors:** Gonzalo Rodríguez-Canosa, Jaime del Cerro Giner, Antonio Barrientos

**Affiliations:** Robotics and Cybernetics Research Group. Centre for Automation and Robotics (CAR UPM-CSIC), Universidad Polité)cnica de Madrid, C/ José Gutierrez Abascal, 2, Madrid 28006, Spain; E-Mails: j.cerro@upm.es (J.C.G.); antonio.barrientos@upm.es (A.B.C.)

**Keywords:** DATMO, multirobot, critical infrastructure surveillance

## Abstract

The detection and tracking of mobile objects (DATMO) is progressively gaining importance for security and surveillance applications. This article proposes a set of new algorithms and procedures for detecting and tracking mobile objects by robots that work collaboratively as part of a multirobot system. These surveillance algorithms are conceived of to work with data provided by long distance range sensors and are intended for highly reliable object detection in wide outdoor environments. Contrary to most common approaches, in which detection and tracking are done by an integrated procedure, the approach proposed here relies on a modular structure, in which detection and tracking are carried out independently, and the latter might accept input data from different detection algorithms. Two movement detection algorithms have been developed for the detection of dynamic objects by using both static and/or mobile robots. The solution to the overall problem is based on the use of a Kalman filter to predict the next state of each tracked object. Additionally, new tracking algorithms capable of combining dynamic objects lists coming from either one or various sources complete the solution. The complementary performance of the separated modular structure for detection and identification is evaluated and, finally, a selection of test examples discussed.

## 1. Introduction

Surveillance of large “structures” is a major modern concern for governments and companies. Energy production centers, transportation infrastructures, food and water supply centers or storage facilities of sensitive classified materials are examples of critical infrastructures (CIs) where a robotic security solution can be applied. In this and other related areas, detection and tracking of dynamic objects (DATMO) has become an emerging research field in which solutions are required for the correct development of multidisciplinary applications, such as traffic supervision [1], autonomous navigation of robots and other vehicles [2,3] or autonomous surveillance of large facilities [4]. Due to this increasing interest, robotic solutions for security and surveillance are currently being developed by different research laboratories [5,6] and commercial enterprises such as MoviRobotics or Robowatch. Although the commercial products have experienced significant improvements during the past years, there are still problems not fully resolved in areas, such as robot positioning, or by the detection and tracking of mobile objects. This paper focuses on this latter subject.

In conventional security and surveillance applications, automatic systems are capable of detecting movement within a surveillance zone, leaving to the human operator the definition of the risk level. Emerging new applications require autonomous surveillance systems capable of both detecting moving objects simultaneously and tracking their trajectories within large security zones. Different sensors, such as laser systems, visual and infrared cameras or ultrasound systems, can be used to detect dynamic objects within a security perimeter. It is the aim of the present work to develop a series of algorithms capable of handling several detected parameters to enable an autonomous decision made by surveillance robots operating in real scenarios. This requires the implementation of accurate methods of detecting and tracking dynamic objects at long distances.

### 1.1. Detection of Dynamic Objects

Most utilized systems for the detection of dynamic objects rely on either video cameras coupled with computer vision, laser imaging detection and ranging sensors (LiDAR) [7,8] and, more recently, time of flight (ToF) cameras [9] or 3D LIDAR [10]. The use of visual or infrared video cameras for DATMO has been proposed for different applications, in which the incorporation of specific data handling methodologies is usually required to improve recognition [11–14]. Other methods based on ultrasonic or infrared sensors are capable of detecting movement in a given area, but not of determining the location or any other feature of the moving object [15]. In another recent approach, sound detection by using a microphone array has been proposed [16].

Laser-based procedures may incorporate different numbers of sensors and rely on specific methods of data analysis. Traditionally, most LiDAR-based applications work with enhanced 2D information, *i.e.*, the sensor provides the depth to all elements in a single horizontal plane. The main difficulty for the analysis is to separate the sensor measurements changes produced by the movement of the robot from the modifications induced by dynamic objects in the environment. To overcome this problem and effectively detect mobile objects, Bobruk and Austin [17] proposed a method in which they compare consecutive laser scans and compensate for the movement of the robot with a fusion between pure odometry data and a translation and rotation produced by an iterative closest point (ICP) algorithm. Another methodology proposed by Chen *et al.* [18] was based on simultaneous location and mapping (SLAM) techniques to generate maps and extract dynamic objects by comparing the static object map with the new additions. Another related approach relies on the use of a conditional particle filter to detect persons in motion [19].

These previous methods do not use 3D information as input for their calculations, a restriction that limits their use for security applications, as they only detect moving objects at a predetermined height. This shortcoming was released by Tanner and Hartmann [9] by using a single time of flight (ToF) indoor camera. In the same line, Swadzba *et al.* [20] were able to track dynamic objects to reconstruct a static scene by using a ToF camera and a 6D data representation consisting of 3D sensor data and computed 3D velocities. Other options combine a 2D LIDAR scanner with a vertical servo to obtain 2.5D data of the environment (range images or point clouds). Using this combination, Ohno *et al.* [21] were able to eliminate the moving objects from the scans of static scenes by comparing collision distances in the same area. More recently, Moosmann and Fraichard [22] have proposed a method consisting of deriving a dense motion field based exclusively on range images for performing object-class independent trajectory estimations.

However, none of the previous approaches use full environment range images to effectively detect and track multiple dynamic objects from multiple robots. Herein, we develop two methods to detect moving objects from a robotic platform using range images. The first one is intended for static platforms and the second for dynamic ones. Furthermore, detection based on this type of data is followed by an effective tracking process using the generated dynamic objects lists.

### 1.2. Tracking of Dynamic Objects

There are several possibilities for tracking dynamic objects using a single robot. One of the most successful approaches [17] uses parameters, such as size and position, in a blob segmentation algorithm to characterize each detected object. These blobs are managed by creating a movement hypothesis with specific position and velocity data for each object. Each hypothesis is stored and updated with the estimated position and velocity of the objects, as well as with a weighting probability of the actual tracking of the moving object.

In multi-robot systems, the information generated by each robot must be combined to enable better tracking. Stroupe *et al.* [23] proposed two-dimensional Gaussian distributions to represent each observation of the object and a statistical procedure based on the Bayes rule and Kalman filters to combine two measurements. Another cooperative target tracking approach as proposed by Wang *et al.* [24] consists of a distributed Kalman filter to estimate the target position. Mazo *et al.* [25] proposed a hierarchical algorithm to locate and track a single dynamic object from data provided by a two-robot system. The two robots share their sensor information to estimate the position of the object by triangulation. Although the problem was efficiently solved for one object, the method is not applicable to a multiple-object environment, because all the robots need to detect and track the same object simultaneously. The main drawback of these methods, intended to detect and track a single object by its simultaneous observation by several robots, is that they cannot be extrapolated to detect and track multiple objects.

The problem of simultaneously tracking several objects has been addressed in the work of Chau *et al.* [26], in which multiple object tracking is achieved by using a multiple features similarity methodology comparing color images. Multiple object tracking using multiple cameras for surveillance applications has been addressed by Kachhava *et al.* [27]. A procedure for tracking multiple walkers with multiple robots equipped with 2D LIDAR sensors has been recently proposed by Tsokas *et al.* [28].

Another possibility for tracking multiple targets would be the use of particle filters [29,30] to combine observations from multiple robots, increasing in that way the quality of the tracking. This technique shows several advantages over other estimators; it is certainly well suited to accommodate the types of uncertainty that arise in the distributed surveillance scenario and allows for estimating future states. Nevertheless, although the previously referred solutions show an important reduction in the bandwidth required by reducing the number of particles exchanged among the robots, the amount of information and the re-simulation requirements are considerable higher than proposed in this work. Moreover, in CIs, it is not common to have multiple robots surveilling the same area, since they are usually equally spread through the whole installation.

In general, the previous methodologies tightly couple detection and tracking, making the latter quite dependent on the type of data and environment set-up requirements used for the detection algorithms. This makes that alternative detection methods not able to be easily integrated together with the tracking process.

### 1.3. Objectives and Scope of the Work

**Figure 1.**
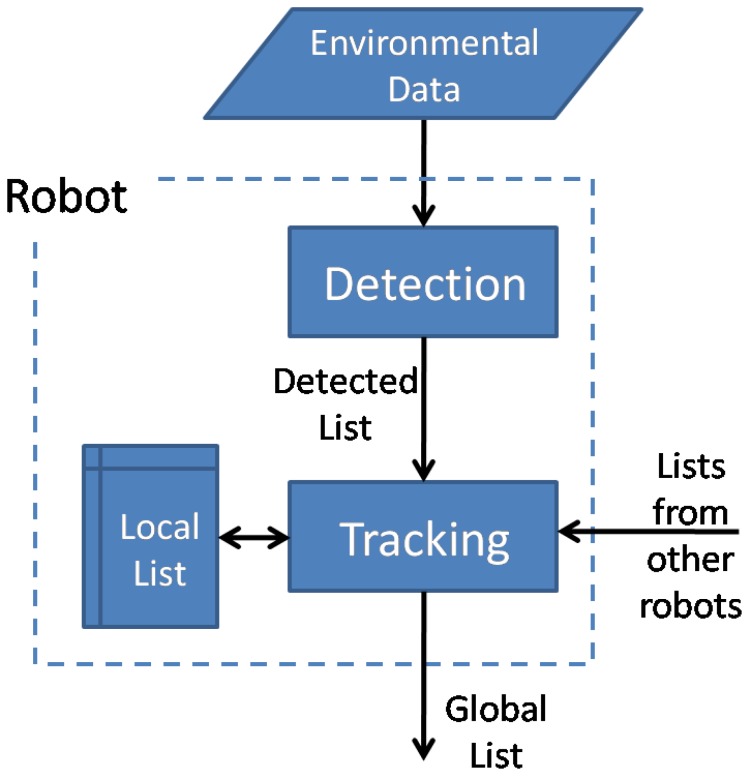
Scheme of the modular concept developed for detection and tracking of mobile objects (DATMO) with multiple robots. Detection and tracking modules work in an autonomous manner.

The method proposed in the present work tackles the previous problems by developing independent detection and tracking algorithms that can work either separately or coupled sequentially in a common DATMO process. The advantage of this approach is that alternative detection algorithms might be used in combination with the tracking algorithms. An example of this capability applied to images taken from an unmanned aerial vehicle (UAV) has been recently proposed by the authors, Rodríguez-Canosa *et al.* [31]. Therefore, in the present work, we develop tracking procedures that are compatible with data supplied by different detection algorithms. This would enable one to apply the tracking process to multiple heterogeneous robots with different sensor configurations. A scheme of the modular concept intended in the present work is shown in Figure 1.

To make the detection algorithms compatible with the tracking, they must provide a list of dynamic objects with at least the following characteristics: a 3D position in a global reference frame and a specific size estimate, both types of data with provided error assessments. If they were available, the tracking algorithms can also process other object properties, such as color, temperature, *etc*. Furthermore, if the detection algorithms were to be applied for surveillance purposes, some common requirement will be that they have to analyze vertical and horizontal environmental data (*i.e.*, at least 2.5D range images, point clouds, stereo images, *etc.*), with a long-distance detection range and a high reliability at medium distances. The detection algorithms developed in the present work comply with all these requirements.

The tracking algorithms developed in the work are intended for surveillance in large outdoor facilities and need to comply with a set of additional requirements, such as a multiple targets/multiple robots tracking, efficient occlusions handling and, as already stated, independence from the detection sensors and procedures. Other important requirements for their practical implementation are that they can work with a decentralized system structure and are easily scalable to work with a variable number of robots. Moreover, although the developed approach depicting a decentralized structure, in which all robots communicate between each other, can be implemented both in a centralized or a decentralized system. To our knowledge, this comprehensive handling of the problem has not been previously considered.

In summary, the paper provides a new modular approach for detection and tracking that allows both centralized and decentralized methods be used in multirobot systems. The modular architecture provides the system with flexibility, allowing total or partial substitution of the algorithms without additional changes. Therefore, it is important to highlight that the work is neither focused on performing a global comparative analysis of DATMO techniques nor in considering planning strategies for task assignment among robots.

Moreover, the software presented in this work has been developed under open source policy and ROS (robotics operative system) premises. Therefore, both detection and tracking algorithms have been implemented as ROS modules. Currently, the authors are working on documenting the code in order to contribute to the ROS official repository. Anyway, up to that moment, the software could be provided by the authors on demand.

The paper is organized as follows. Section 2 describes the algorithms developed for detecting moving objects. Section 3 presents the developed tracking algorithms. Section 4 shows a selection of tests. The conclusions of the work are presented in Section 5.

## 2. Detection of Dynamic Objects

In this section, we firstly present the type of input data used by the two algorithms developed to detect moving objects. Then, we describe these two detection algorithms and their respective use with static and dynamic platforms.

The developed algorithms are designed to use 2.5D range images (an image with depth information in each of its pixels) as input data from the environment. This data can be originated from different sensors, such as a time of flight (ToF) camera or a 3D LIDAR. Another common combination is a 2D LIDAR scanner mounted on a pan-tilt unit.

The data matrix obtained is called the distance matrix (*D*), where each element represents a distance to a collision point. For each matrix element (*i, j*), there are three known parameters: the distance, *D_ij_*, and the angles formed by the beam with respect to the laser coordinate frame.

**Figure 2.**
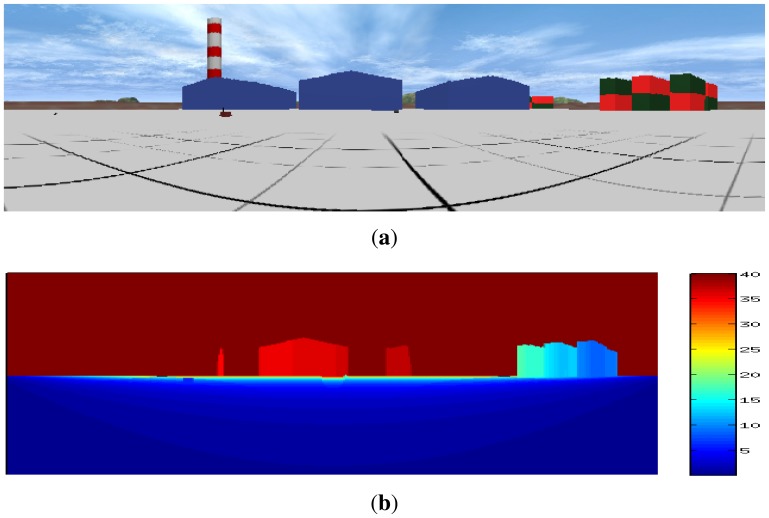
Example of a color (a) and range image (b) of the same scene.

To produce this type of range image, ToF cameras (such as the Kinect^®^) are becoming very popular, because they provide range information together with color information without arduous calibration processes. However, when using other sensors, such as 2D or 3D LIDAR devices, sensor fusion techniques [32] can be used to assign color information to the range data matrix (*D*). An example of this sensor fusion can be seen in Figure 2.

### 2.1. Detection from a Static Platform

The first developed algorithms aim at detecting moving objects in the environment from a sequence of 2.5D range images. Since the sensor is static, any change in the images is produced by dynamic objects or measurement noise. The main problem is to separate these two effects.

The developed procedure firstly determines the difference between two consecutive range images. Then, the distance differences that exceed a predefined threshold are eliminated according to the criteria specified in Equation (1).

(1) $$\text{Dif}(i,j)=\{\begin{array}{cc} 1 & \text{if}\left|D_{ij}^{k}-D_{ij}^{k-1}\right|>Dif_{\text{lim}}\\ 0 & \text{if}\left|D_{ij}^{k}-D_{ij}^{k-1}\right|<Dif_{\text{lim}} \end{array}$$

In this equation, $D_{ij}^{k}$ is the current distance matrix, $D_{ij}^{k-1}$ the distance matrix previous to the current one, *Dif*(*i, j*) the differential distance matrix and *Dif_lim_* the threshold value used to extract the points where there is movement. This threshold value takes into account the resolution and the accuracy of the sensor and is usually set at a value 25%–50% higher than the accuracy.

For a Hokuyo UTM-30LN laser with an accuracy of ±50 mm in a 30-m range, this threshold was set at 7–8 cm.

**Figure 3.**
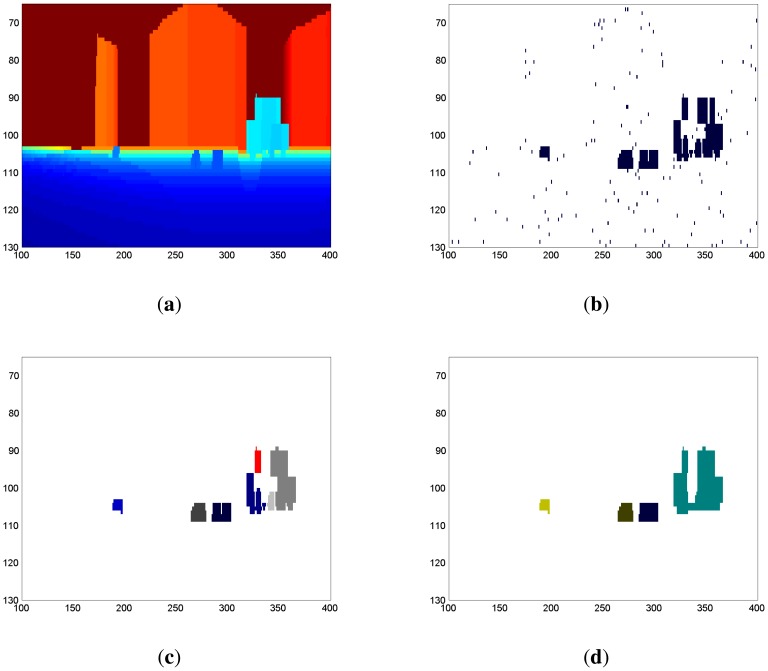
Laser matrices along the detection procedure. Plots (**a**) represents a distance matrix; (**b**) represents the difference between two consecutive distance images; (**c**) is the filtered difference; and (**d**) the objects with more than four points after the erosion and dilation process.

The results of this calculation can be seen in Figure 3b, representing a matrix in which the nonzero points indicate movement. Since not all nonzero points necessarily belong to dynamic objects, a eight-connectivity grouping process is carried out to isolate and remove the points caused by measurement errors (Figure 3c). The resulting groups are labeled as differentiated objects, provided that they contain more than four points. Otherwise, they are discarded. The discarded groups usually result from measurement errors or small changes in the environment. At this stage, a matrix is obtained in which all the pixels belonging to the same object are marked with the same label and in which the other pixels are zero. A representation of this matrix can be seen in Figure 3c, in which each detected object is highlighted with a different color.

A problem with the eight-connectivity grouping technique is that, if object surfaces are homogeneous to the sensor, a single moving object can be detected as multiple separated fragments. To merge these fragments into the real object, two artificial vision techniques, dilation and erosion, are applied. The result of applying these techniques to the process is shown in Figure 3d.

The main advantage of this method with respect to other approaches in the literature [18] is that, by using range images, it does not limit the detection of dynamic objects to a plane, and therefore, it may provide much more accurate information about the environment.

#### 2.1.1. Dynamic Reference Range Image for Environment Perception

**Figure 4.**
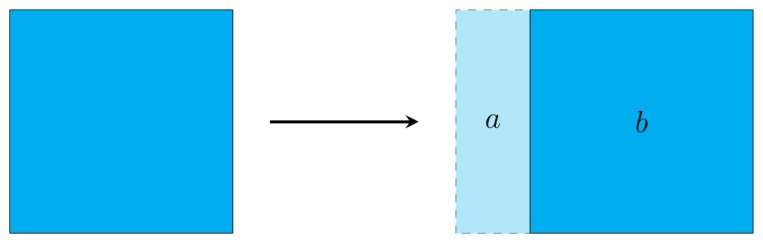
Scheme for the detection of a moving square. (**a,b**) The area marked as object in the differentiation matrix, even if the laser measurements (**a**) correspond to the environment. The reference matrix is used to isolate the points belonging uniquely to the object, area (**b**).

According to the criteria defined in Equation (1), a group of matrix points could be identified as part of the moving object even though the laser measurements actually correspond to the environment. Figure 4 illustrates the nature of this problem with the detection of a moving square. Area (a) includes those points that were part of the object in a previous distance matrix, but no longer form part of it after its movement to the right. However, according to Equation (1), if the difference between points of the distance matrix is larger than a defined threshold value, points in (a) will be marked as part of the object.

To eliminate this incongruence and effectively identify and remove these points, a dynamic reference matrix $\left(D_{ij}^{\text{ref}}\right)$ is recursively updated with each range image. $D_{ij}^{\text{ref}}$ is kept updated along successive iterations according to the mean $\left({\bar{D}}_{ij}^{k}\right)$ and variance $\left(V_{ij}^{k}\right)$ values of the last three range distance images (Equation (2)). The slower the objects move, the higher the number of range images that must be taken into account for an accurate perception of the environment.

(2) $$\begin{array}{c} {\bar{D}}_{ij}^{k}=\frac{1}{3}{\text{∑}}_{l=0}^{l=2}D_{ij}^{k-l}\\ V_{ij}^{k}=\frac{1}{3}{\text{∑}}_{l=0}^{l=2}\;{\left(D_{ij}^{k-l}-{\bar{D}}_{ij}^{k}\right)}^{2} \end{array}$$

Updating results from the application of two rules (Equation (3)) to all the distance matrix points: (i) if the calculated variance is smaller than the reference variance, the values are updated; (ii) if the variance is smaller than four times the reference variance and the calculated mean distance is greater than the reference distance, the values are also updated.

(3) $$\begin{array}{r} \text{If}\;V_{ij}^{k}<V_{ij}^{\text{ref}}\\ \text{If}\;V_{ij}^{k}<4V_{ij}^{\text{ref}}\&{\bar{D}}_{ij}^{k}>D_{ij}^{\text{ref}} \end{array}\}\begin{array}{c} D_{ij}^{\text{ref}}={\bar{D}}_{ij}^{k}\\ V_{ij}^{\text{ref}}=V_{ij}^{k} \end{array}$$

After this evaluation, larger distances are generally maintained in $D_{ij}^{\text{ref}}$, as they likely belong to the background. Small distance values (generally due to objects close to the scanner) could also substitute reference distances if the concerned objects remain static for a certain period of time. This procedure is also useful to incorporate those dynamic objects that have ceased to move for a long period of time and, therefore, have become a static part of the environment with respect to the reference matrix.

#### 2.1.2. Real Characteristics of Dynamic Objects

Once the dynamic objects have been detected, it is necessary to extract their defining characteristics, in this case, position, size and color. For this purpose, a collision matrix containing the collision points in a global reference system is calculated. This collision matrix or point cloud might be calculated at the beginning of the algorithm together with the whole distance matrix, although the overall process is much faster when it only involves the object points.

This method can be considered as the core of the static detection algorithm to determine the position and size of the objects. It proceeds along the following steps:

1. Determination of dynamic points. For each element (*i, j*) in each previously labeled object, it compares *D_ij_* with $D_{ij}^{\text{ref}}$ and removes those points (*i, j*) with values similar to those in the reference.
2. Calculation of position. It calculates a central cell in the range matrix for object *O_k_*. The collision point of this cell in the global reference system is taken as the position of the object. The obtained position is the center of the object side facing the sensor. The depth of the object cannot be estimated, as no depth information is available.
  (a) $i_{\text{cent}}^{O_{k}}={\text{∑}}_{\forall i\in O_{k}}\;i/n_{\text{row}}$
  (b) $j_{\text{cent}}^{O_{k}}={\text{∑}}_{\forall j\in O_{k}}\;j/n_{\text{col}}$
3. Calculation of size. For all object points, the maximum and minimum values of each coordinate are calculated. Size is estimated by the difference between these two values. Since the input data are 3D points distributed on the side of the object facing the sensor, only two dimensions would be significant, and the maximum value from the three calculated dimensions is kept.
  (a) *Size* = *max*([*max*(*x*) − *min*(*x*), *max*(*y*) − *min*(*y*), *max*(*z*) − *min*(*z*)])

After repeating this process for all objects, a list that contains the positions and sizes associated with all of them is obtained. These steps are summarized in Algorithm 1.

**Algorithm 1** Static Detection Algorithm.
**Require**: Distance matrices 
$D_{ij}^{k}$ and 
$D_{ij}^{k-1}$. Update 
$D_{ij}^{\text{ref}}$ with Equation (3). Differentiate 
$D_{ij}^{k}$ and 
$D_{ij}^{k-1}$ according Equation (1). Eight-connectivity grouping of pixels. Dilation and erosion. Position and size calculation. **return** Object list *L_k_*.


### 2.2. Detection from a Dynamic Platform

When the data proceeds from a sensor on a moving robot, the coordinate frame from the measurement changes as the robot moves. Thus, the previous approach of range images differentiation is not applicable. To avoid this restriction, a comparison of ground reconstructions to determine the areas containing moving objects is proposed. Since this procedure deals with point clouds and provides the actual heights of buildings, dynamic objects and other elements in the environment, it is expected to be more reliable than other available terrain-reconstruction approaches using a single predefined height for all features [33].

A ground reconstruction is a multiple layer cell map in which different values are assigned to each cell. These values are obtained from the points of the point cloud whose projections on the ground are inside each corresponding cell. The ground reconstruction comparison is carried out for maps with confidence, maximum height and X,Y gradient layers. The main parameters of these maps are:

1. Cell size: This stands for the resolution of the map. This parameter also defines the smallest object that the system is able to detect. Decreasing the size of the cell would increase the precision of the detection system, but will increase the computational cost. This minimum size is also limited by the sensor resolution.
2. Height layer: This parameter determines the characteristic height assigned to each cell. Differences between two consecutive reconstructions indicate movement. We employ two different formulations of the height: (i) the mean height and (ii) the maximum height. Maximum heights turned out to be more reliable for detecting dynamic objects.
3. Gradient layers: These are used to estimate the slopes of the terrain. Gradients are calculated along fixed axes using only the collision points within the same cell. This parameter is used to discard static elements, such as walls, fences, *etc*. When a robot comes closer to one of these elements, the maximum height of the cell can be increased, although the gradient in the cell remains constant, thus discarding the designation of such cells as dynamic objects.
4. Confidence layer: This is used to measure the reliability of the data stored in each cell. Since laser scans have a high horizontal resolution, cells will usually have multiple collision points. Two different methods to calculate the confidence have been tried. The first one utilizes the number of impact points and the second the dispersion of the impact points inside each cell. The latter procedure provides more reliable results.

**Figure 5.**
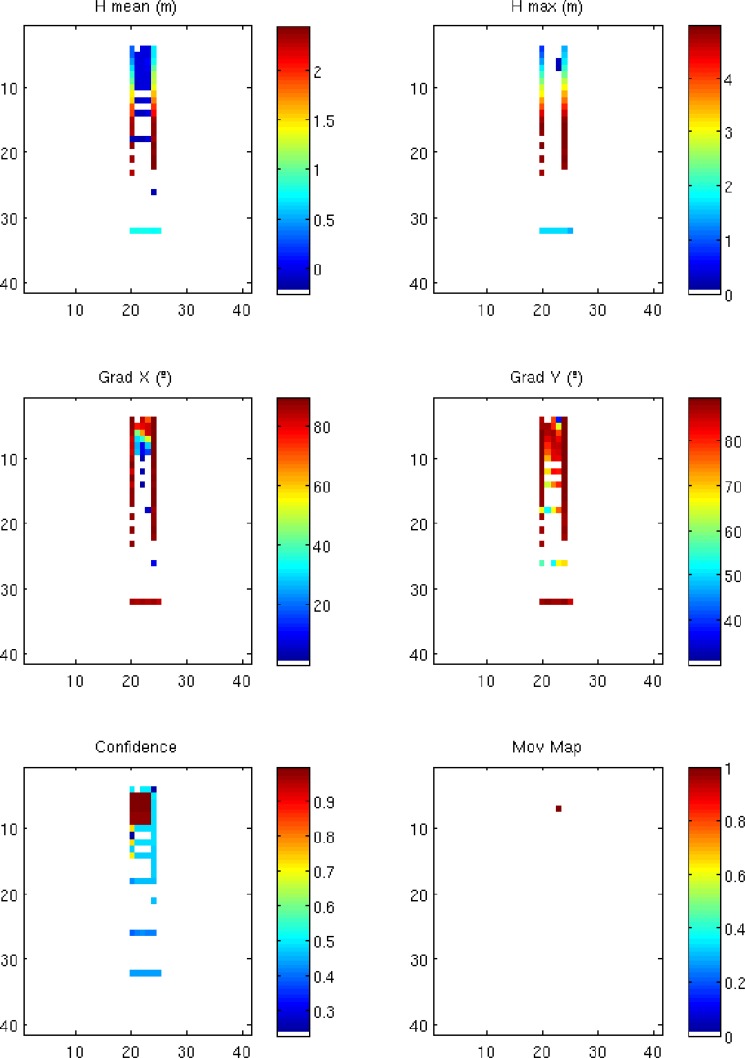
Ground reconstruction example of a robot that, passing between two walls, is detecting a mobile object.

An example of this map can be seen in Figure 5, where a portion of the map generated by a robot that is moving between two walls is shown.

### 2.3. Map Comparison

Since, in this case, the processed measurements come from a sensor mounted in a moving robot, the change in the robot coordinate frame has to be taken into account. One option would be to keep increasing the map size as long as the robot moves. With this approach, the computational processing cost increases with the dimension of the map. The second approach consists of changing the map center in the global coordinate frame each time that the range of the sensor reaches the limit of the map.

**Figure 6.**
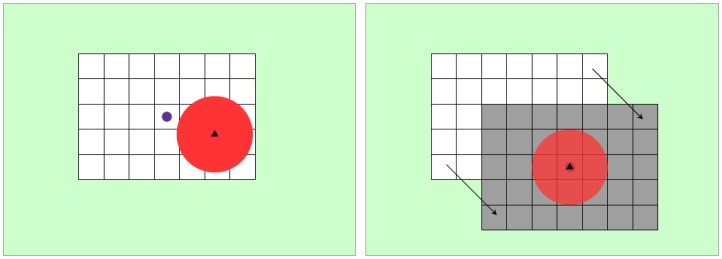
Movement of the center of the cell map when the laser range exceeds its dimensions. The robot position is indicated by a black triangle and the laser range with a red circle. When the laser range exceeds the cell map dimensions (white rectangle), the cell map is relocated, as indicated by the arrow.

At the beginning of the detection process, the center of the map is taken at the position of the robot and the dimensions of the map to at least encompass 150% of the detection range in both directions. When the robot moves and the detection range exceeds the map dimensions, the center of the map must be changed, as shown in Figure 6. When this occurs, the detection process is paused during three iterations to enable that the reconstruction maps with reference to the same center and can be properly compared.

This technique requires a trustworthy navigation system in order to estimate the movement of the robot. Thus, a classical combination for outdoor robot positioning techniques, such as odometry and GPS, provides a precise and robust navigation solution. An additional compass is also available in order to improve the dynamic estimation of the yaw angle. It is worth noting that big infrastructures usually rely on their own GPS base stations, which allows for obtaining precisions of several centimeters in real time by using real time kinematics (RTK) corrections.

Another widely used technique for estimating the movement of the robot could be the use of visual odometry. The performance of this technique highly depends on changes in light or soil texture. Moreover, considering that robots should be able to operate in poor light conditions or even in dark, a solution based on a combination of global navigation satellite system and inertial sensors (GNSS-INS) is considered more robust. However, the integration of visual odometry into in the navigation system would provide the system with a higher robustness in situations in which the GPS signal is not available.

Any particular range distance image or point cloud yields a ground reconstruction cell map (*R_k_*). When two consecutive reconstructions (*R_k_* and *R_k_*_−1_) are compared, a movement map (*M_k_*) is obtained. The following rules are applied to perform this process:

1. The confidence of each cell must be greater than a predefined threshold value; usually 0.5.
2. The difference between gradients X and Y must be larger than zero and smaller than a certain threshold value defined to get rid of static elements.
3. The difference between the maximum heights must be larger than a certain value. Dynamic objects smaller than the defined threshold value would not be detected.

When two consecutive movement maps (*M_k_* and *M_k_*_−1_), obtained from three consecutive reconstructions with the same center, are available, they are compared to eliminate possible false detections. Each cell is evaluated independently. For the effective movement map in each iteration (*Moυ_k_*), a cell is marked as positive (i.e., meaning that there is movement in this cell) if:

1. There is movement in the current movement map. *M_k_*(*i, j*) = 1
2. There is movement in the environment around the same cell in the previous map. *M_k_*_−1_(*u*, *υ*) = 1; *u* = [*i*, *i* + 1, *i* − 1]; *υ* = [*j*, *j* + 1, *j* − 1]

Once an effective movement map (*Moυ_k_*) has been obtained, an eight-connectivity grouping of the cells is carried out. Each group of cells represents an object with the same position as the center of the group. In this case, size estimation is not straightforward, because discriminating, in the same cell, the points belonging to the object from the points of the environment is not straightforward. As an alternative, a size estimation based on the number of cells occupied by each object is carried out, although the result is slightly less accurate than in the static detection case.

At the end of this process, we obtain a list of dynamic objects and their associated positions. These steps are summarized in Algorithm 2.



**Algorithm 2** Dynamic Detection Algorithm.
**Require:** Distance matrices *D_k_*, *D_k_*_−1_ and *D_k_*_−2_ Calculate reconstruction *R_k_*, *R_k_*_−1_ and *R_k_*_−2_**Ensure:** Center of reconstructions is the same. Calculate movement maps *M_k_* and *M_k_*_−1_. Compare *M_k_* and *M_k_*_−1_ to obtain *Moυ_k_*. Eight-connectivity grouping of cells. Calculate position and size of objects. **return** Object list *L_k_*


## 3. Tracking of Dynamic Objects

In this section, we propose a tracking procedure that can work with dynamic object data supplied by any kind of detection algorithms complying with the conditions specified in Section 2. Another singularity of the method is that it can also handle and process dynamic object lists provided by other robots or sensors working in the same surveillance area.

Typically, position and size and their corresponding errors are the main parameters associated with each object. All tests and evaluations in this work have been carried out with these input parameters. However, our algorithms might also manage additional information from the detected objects provided by other sensors, such as color or temperature. This additional information might be assigned to each pixel by using sensor fusion techniques [34].

### 3.1. Kalman Filter for Tracked Objects

In the present approach, each robot handles a different list of tracked objects, and a single Kalman filter is used to track and predict the state of each object.

The normal formulation of a Kalman filter [35] implies a lineal dynamic model, where each state, *k*, depends on the previous state, as shown in Equation (4), where *x_k_* is the current state, *A_k_* is the state transition model, *x_k_*_−1_ the previous state, *B_k_* the control input model, *u_k_* the velocities vector and *w_k_* the process noise, which is assumed to be a multi-variant normal distribution with a zero mean and a *Q_k_* covariance (*w_k_*(*t*) ∼ *N*(0, *Q_k_*(*t*))).

(4) $$x_{k}=A_{k}\cdot x_{k-1}+B_{k}(t)\cdot u_{k}+w_{k}(t)$$

Each dynamic object has a set of associated features that are considered its state. This state encompasses its position in the global reference system and some physical features, such as size and color, as shown in Equation (5). Other parameters, such as temperature, could also be added to the object state if provided by the detection system.

(5) $$x_{k}={\left[x,y,z,\text{size},R,G,B\right]}^{\prime }$$

Using a constant velocity model, the matrices from Equation (4) are shown in Equation (6), where Δ*t* = (*t_k_* − *t_k_*_−1_) is the time difference between the current state and the predicted state. This way, we consider *u_k_* to be the velocity of the object, assuming that both the size and the color remain constant.

(6) $$\begin{array}{ll} A_{k}={\text{I}}_{7}=\left[\begin{array}{lllllll} 1 & 0 & 0 & 0 & 0 & 0 & 0\\ 0 & 1 & 0 & 0 & 0 & 0 & 0\\ 0 & 0 & 1 & 0 & 0 & 0 & 0\\ 0 & 0 & 0 & 1 & 0 & 0 & 0\\ 0 & 0 & 0 & 0 & 1 & 0 & 0\\ 0 & 0 & 0 & 0 & 0 & 1 & 0\\ 0 & 0 & 0 & 0 & 0 & 0 & 1 \end{array}\right], & B_{k}(t)=\left[\begin{array}{lll} \Delta t & 0 & 0\\ 0 & \Delta t & 0\\ 0 & 0 & \Delta t\\ 0 & 0 & 0\\ 0 & 0 & 0\\ 0 & 0 & 0\\ 0 & 0 & 0 \end{array}\right] \end{array}$$

The process noise is also time dependent, as shown in Equation (7)

(7) $$Q_{k}(t)=\left[\begin{array}{lllllll} \Delta t^{2}\sigma_{x}^{2} & 0 & 0 & 0 & 0 & 0 & 0\\ 0 & \Delta t^{2}\sigma_{y}^{2} & 0 & 0 & 0 & 0 & 0\\ 0 & 0 & \Delta t^{2}\sigma_{z}^{2} & 0 & 0 & 0 & 0\\ 0 & 0 & 0 & \sigma_{\text{size}}^{2} & 0 & 0 & 0\\ 0 & 0 & 0 & 0 & \sigma_{\text{RGB}}^{2} & 0 & 0\\ 0 & 0 & 0 & 0 & 0 & \sigma_{\text{RGB}}^{2} & 0\\ 0 & 0 & 0 & 0 & 0 & 0 & \sigma_{\text{RGB}}^{2} \end{array}\right]$$

The process noise, *Q_k_*, represents the variation between the real value of the object characteristics and the estimate obtained from the sensors. Due to its time dependency, it has to be set in each iteration. Based on sensor features, the values ( $\sigma_{x}^{2}$, $\sigma_{y}^{2}$, $\sigma_{z}^{2}$, $\sigma_{\text{size}}^{2}$, $\sigma_{\text{RGB}}^{2}$) must be defined before the system is initialized.

For the correct behavior of the tracking module, it is very important that a reasonable movement model is chosen for the tracked object. There are several options to make these predictions, which are based on constrained networks [36], on learned motion patterns [37] or on statistical approaches [38]. The herein applied approach does not require any previous knowledge about the object (since it is not available), but previous observations made by the detection algorithms. A constant linear and angular velocity model has been chosen for the ground coordinates (*x*, *y*) and a constant linear velocity model for the altitude. This latter assumption is justified, because most objects would be ground-bounded and most changes in height will be produced by errors in the detection process.

**Figure 7.**
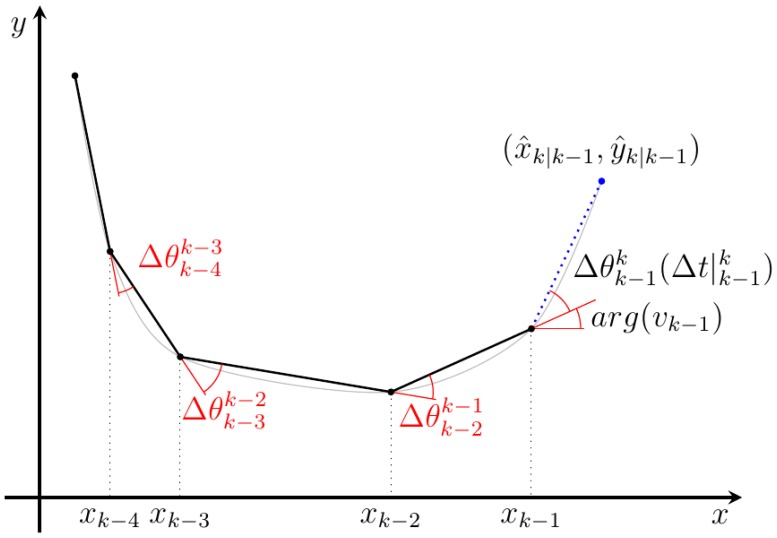
Velocities calculation diagram. The different detected positions of a dynamic object are connected by the angle increment used to obtain the marked angular velocity. In the velocity calculation, both the linear and angular velocities are used to improve the movement model.

To properly make this prediction, the method computes the current velocity by using a programmable number of historical records (*n*), according to Equation (8). The involved angles are shown in Figure 7.

(8) $$\begin{array}{ll} \bar{\omega }=\frac{1}{n-1}\overset{k-1}{\underset{i=k-n}{\text{∑}}}\frac{\Delta \theta |\begin{array}{l} i+1\\ i \end{array}}{\Delta t|\begin{array}{l} i+1\\ i \end{array}}; & \bar{\upsilon }=\frac{1}{n-1}\overset{k-1}{\underset{i=k-n}{\text{∑}}}{\left|\upsilon \right|}_{i} \end{array}$$

To incorporate the calculated mean velocities in the prediction equation, they have to be expressed according to the coordinates (*υ_x_*, *υ_y_*) obtained according to Equation (9).

(9) $$\begin{array}{l} \upsilon_{x}=\bar{\upsilon }\cos(\bar{\omega }\Delta t{|}_{k-1}^{k}+\arg(\upsilon_{k-1}))\\ \upsilon_{y}=\bar{\upsilon }\sin(\bar{\omega }\Delta t{|}_{k-1}^{k}+\arg(\upsilon_{k-1})) \end{array}$$

To simplify the prediction process and allow the use of a linear Kalman filter, but still be able to predict curvilinear trajectories, the information of the lasts positions is used to calculate an expected velocity based on the last position of the object.

### 3.2. Single Robot Object Tracking

The tracking process requires an efficient and robust pairing procedure capable of identifying the objects coming from the detection algorithms among the objects in the list stored locally in each robot. Such a pairing is carried out by comparing the predicted state (*x̂*_*k*|*k*−1_) and the new obtained measurements $\left(z_{k}^{i}\right)$. When the object measurements come from the detection module in the same robot, all of them are characterized by a unique time.

The pairing process is based on the Mahalanobis distance (Equation (10)) [39], a well-known function that determines the similarities between two multidimensional variables.

(10) $$D_{M}({\hat{x}}_{k|k-1}^{i},z_{k}^{j})=\sqrt{\overset{N_{\upsilon }}{\underset{\upsilon =1}{\text{∑}}}\frac{{\hat{x}}_{k|k-1}^{i}{\left(\upsilon \right)}^{2}-z_{k}^{j}{\left(\upsilon \right)}^{2}}{P_{k|k-1}^{i}(\upsilon )}}$$

After determining all possible pairs, those with the smallest distances are selected until completion of the pairing process. The number of selected pairs will be equal to the number of elements in the list with less objects. After the selection of the pairs according to the Mahalanobis distance criterion, the objects in the local list are separated into two subsets to perform the iteration solely with the objects from which the system has received new measured data (*i.e.*, those that have been paired). If the number of objects increased considerably, an alternative method would be implemented in order to avoid the calculation of the distance for all the possible pairs (*i.e.*, k-distance tree).

The selection of this distance threshold is not trivial and will define the performance degree of the algorithm. Additionally, since it is possible that a single moving object is detected as two nearby objects, this threshold will determine whether these duplicates are incorrectly assigned to another object or, on the contrary, are introduced in the objects list to be handled by a filter reduction mechanism.

With these selected pairs, the two lists are divided into three groups:

- $\left({\hat{x}}_{t_{in|k-1}}^{\text{sel}},z_{k}^{\text{sel}}\right)$: The objects included in the selected pairs will perform a Kalman filter (KF) iteration with $z_{k}^{\text{sel}}$ as input.
- ${\hat{x}}_{k-1|k-1}^{\text{no sel}}$: Non-selected objects in *L_loc_*. They will be evaluated by the elimination rules to verify whether they must be removed from the local list.
- $z_{k}^{\text{no sel}}$: New non-paired objects from *z_k_*. They will be introduced in *L_loc_* as a new KF.

#### 3.2.1. Elimination Conditions

Elimination conditions to remove objects from the local list have been incorporated into the algorithm, both to avoid ever-growing lists of dynamic objects and to preclude false pairings produced after long periods of time. The defined rules address two main magnitudes: time and distance.

The first considered parameter is the time elapsed since the last observation of a given object. The condition is directly reflected in the Kalman filter, because the process noise (*Q_k_*, as shown in Equation (7)) is already time dependent. This time condition can be expressed as shown by Equation (11).

(11) $$\text{if}\;(t_{in}-t_{\text{det}})>t_{\text{max}}\;\text{then}\;\text{remove object}$$

The second condition refers to the traveled distance predicted for each object in *L_loc_*. If the filter estimates that the robot has traveled a distance longer than a given threshold, then the object is eliminated from the list, as shown in Equation (12); since a different threshold distance is set for each robot according to its own detection distance. This criterion will be efficient for eliminating those objects that are no longer within its range. It is most likely that fast moving objects are eliminated by this condition, rather than by the previous one referring to elapsed time.

(12) $$\text{if}\;\left\|({\hat{x}}_{t_{in}|k-1}^{\left(x,y,z\right)}-{\hat{x}}_{k-1|k-1}^{\left(x,y,z\right)}\right\|>{\text{dist}}_{\text{max}}\;\text{then}\;\text{remove object}$$

#### 3.2.2. Object Scoring

The elimination of false positives produced by the detection module is carried out in the tracking module by using a scoring mechanism. Thus, the tracking module assigns a score to each object provided by the detection module. Only objects with a score higher than a threshold are considered as “real” dynamic objects and, therefore, are communicated to other robots or to the central station. Table 1 presents the scoring mechanism for the three possible situations in which the score of a given object will change. This threshold has been experimentally set to four, although this limit will depend on the frequency of the detection algorithm and on the type and range of the sensors. Thus, the faster the detection system, the higher the score that should be used in order to eliminate false positives. From a practical point of view, this limit depends on the number of consecutive times that the detection module has to detect the object, so as to be included in the list of dynamic objects. Once a dynamic object is marked as a real object, the scoring procedure losses its purpose, because the elimination rules do not depend on this particular score (see Section 3.2.1.).

The complete single robot object tracking process is described in Algorithm 3.



**Algorithm 3** Single Robot Object Tracking Algorithm.
**Require:** Input object list with *n_in_* objects.**Require:** Local object list with *n_loc_* objects.**Ensure:**
*n_in_* > 0 ∨ *n_loc_* > 0.1:**for**
*i* ∈ *L_in_*
**do**2: **for**
*j* ∈ *L_loc_*
**do**3:  Δ*t* = *t_in_* − *t_det_*(*j*).4:  Temporal Kalman filter prediction with object *j* and Δ*t*.5:  Mahalanobis distance according to Equation (10)6:  Possible pairs distance matrix *P_D_*[*i*, *j*] = *D_M_*(*i*, *j*).7: **end for**8:**end for**9:Create selected pairs list.10:**while** Size(Selected Pairs)< *min*(*n_in_*, *n_loc_*) **do**11: Select minimum valid value of *P_D_*12: **if**
*P_D_*[*i*, *j*] < *D_M_*_∣_*_max_*
**then**13:  Include pair (*i*, *j*) in selected pairs.14:  Mark row *i* and column *j* with invalid values.15:**else**16:  Stop selecting pairs.17: **end if**18:**end while**19:KF iteration for objects in selected pairs.20:Eliminate objects from *L_loc_*. ▹ Not fulfilling elimination conditions21:Add new objects not paired to *L_loc_*.22:Filter reduction to detect duplicates.23:Update objects scoring.


### 3.3. Multirobot Object Tracking

For a multirobot application, it is necessary to share the dynamic object information among the different robots and the control station. This implies combining the information from two different object lists. List merging will always involve a local list (*L_loc_*) of dynamic objects that must be updated with the information contained in an input list (*L_in_*). There are some restrictions that preclude the direct use of Algorithm 3 to share information between different robots. Since dynamic object lists from different robots have been originated at different times, the Kalman filter prediction stage cannot be applied at the same time to all objects, *L_loc_*. To overcome this problem, a new pairing algorithm (Algorithm 4) has been developed. It has a structure similar to that of Algorithm 3, but it deals effectively with the different time origins. Another important difference is that in this new algorithm, the objects list values in *L_in_* have different covariance values, thus precluding the use of the Mahalanobis distance.



**Algorithm 4** Multirobot Object Tracking Algorithm.
**Require:** Input object list with *n_R_*_1_ objects**Require:** Local object list with *n_R_*_2_ objects**Ensure:**
*n_R_*_1_ > 0 ∨ *n_R_*_2_ > 01:**for all**
*i* ∈ *L_R_*_1_
**do**2: **for all**
*j* ∈ *L_R_*_2_
**do**3:  **if**
$\left|t_{i}^{R1}-t_{j}^{R2}\right|>\Delta t_{\text{max}}$
**then**4:   Mark pair as invalid.5:  **end if**6:  **if**
$\Delta t=t_{i}^{R1}-t_{j}^{R2}\geq 0$
**then**7:   
${\hat{x}}_{k|k-1}^{j}(t_{i}^{R1})=A\cdot {\hat{x}}_{k-1|k-1}^{j}+B\cdot u_{k-1}(t_{i}^{R1}).$8:   Calculate 
$D_{B}({\hat{x}}_{k|k-1}^{j}+{\hat{x}}_{k-1|k-1}^{i})$.9:  **else if**
$\Delta t=t_{j}^{R2}-t_{j}^{R1}>0$
**then**10:   
${\hat{x}}_{k|k-1}^{i}(t_{j}^{R2})=A\cdot {\hat{x}}_{k-1|k-1}^{i}+B\cdot u_{k-1}(t_{j}^{R2}).$11:   Calculate 
$D_{B}\left({\hat{x}}_{k|k-1}^{i},{\hat{x}}_{k-1|k-1}^{j}\right)$.12:  **end if**13:  Possible pair distance matrix *P_D_*[*i*, *j*] = *D_B_*(*i*, *j*).14: **end for**15:**end for**16:Select *min*(*n_R_*_1_, *n_R_*_2_) pairs using lines 9–18 from Algorithm 3 with *D_B_*_∣_*_max_*.17:**for all**
*j* ∈ *L_R_*_2_
**do**18: **if**
*j* ∈ selected pairs (Pair (*i*, *j*)) **then**19:  Update information of object *i* with info from object *j*.20:**else**21:  Add *j* in *L_R_*_1_
**if**
*score_j_* > *score_min_*.22: **end if**23:**end for**


#### 3.3.1. Localization Errors

In the single robot case, the uncertainty in the robot position was not included in the calculation under the assumption that within the same iteration, it was the same for all the objects. However, when lists from different robots have to be combined, this assumption is no longer valid.

The variances of the detected position for dynamic objects can be calculated with respect to the variances in the distance measurements and the variances of the detecting robots' positions. As a result of this calculation, one obtains the position variances defined by Equation (13), where *z^d^* and *z^ϕ^* are the distance and relative angle to the object and *σ_d_* and *σ_ϕ_* the variances of the corresponding measurements.

(13) $$\begin{array}{c} \sigma_{x}^{2}={\text{c}}^{2}\sigma_{d}^{2}+\sigma_{x_{1}}^{2}+{\left(\text{s}z^{d}\right)}^{2}(\sigma_{\phi }^{2}+\sigma_{\theta_{1}}^{2})\\ \sigma_{y}^{2}={\text{s}}^{2}\sigma_{d}^{2}+\sigma_{y_{1}}^{2}+{\left(\text{c}z^{d}\right)}^{2}(\sigma_{\phi }^{2}+\sigma_{\theta_{1}}^{2}) \end{array}\|\begin{array}{c} \text{c}=\cos(z^{\phi }+\theta_{1})\\ \text{s}=\sin(z^{\phi }+\theta_{1}) \end{array}$$

**Figure 8.**
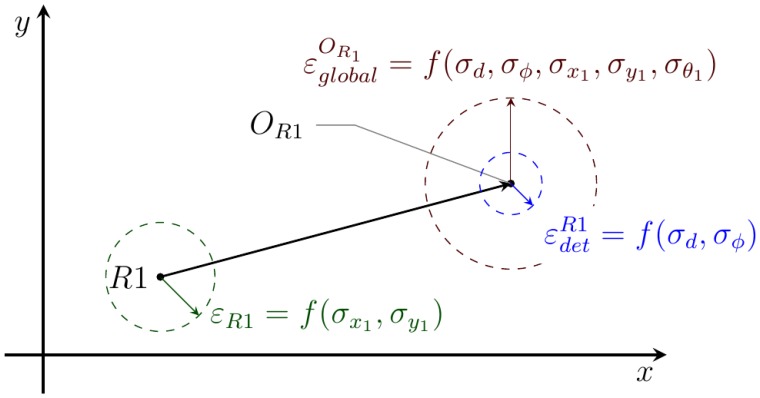
Influence of the robot position error onto the tracked position error. Robot *R*1 with a localization error, *ε_R_*_1_, detects a moving object in position (*O_R_*_1_) with an error, $\varepsilon_{\text{det}}^{R1}$. The global error in the object position $\left(\varepsilon_{\text{global}}^{O_{R1}}\right)$ is a function of the other errors.

Figure 8 shows a schematic diagram explaining why the global error in the object tracked position (*O_R_*_1_) increases when it is expressed with respect to the global coordinate frame $\left(\varepsilon_{\text{global}}^{O_{R1}}\right)$ instead of to the robot position $\left(\varepsilon_{\text{det}}^{R1}\right)$.

A critical problem by any tracking process is to define a procedure to compare two lists of variables with different variances in order to find the most accurate pairs. It has been already mentioned that the Mahalanobis distance cannot be used for this purpose, and therefore, a new metric has to be implemented with the restriction of efficiently handling variables with different covariance values.

When performing the merging of the dynamic lists provided by different sources, the herein proposed algorithm solves the previous problems by taking into account both the actual acquisition times of each set of measurements and the covariance values of these measurements for each object. This means that two dynamic objects lists have to be paired. For this alternative pairing process, it is necessary to calculate a characteristic distance for each possible pair. This distance calculation will depend on the detection time for each object in the pair. For a pair of objects, *i*(*R*1) and *j*(*R*2), representing moving objects tracked by two different robots, three different situations can be envisaged, in which:

- **If** $t_{i}^{R1}>t_{j}^{R2}$ **then** temporal KF prediction of object *j* to $t_{i}^{R1}$.
- **If** $t_{i}^{R1}<t_{j}^{R2}$ **then** temporal KF prediction of object *i* to $t_{j}^{R2}$.
- **If** $\left|t_{i}^{R1}-t_{j}^{R2}\right|>\Delta t_{\text{max}}$ **then** pair is not viable.

It is worth nothing that a significative increment in the time difference will make the information in the local list non-usable. Even if the objects were the same, the new information would be included in the local list as a new object.

The first two situations require a calculated distance to evaluate the pairing by using, in each case, the most recent values for *x* and *z*. We propose the use of the Bhattacharyya [40] distance, a correlation distance that allows a direct comparison between two distributions and their covariance matrices. Equation (14) shows the application of the Bhattacharyya distance to our case by using the available information.

(14) $$\begin{array}{c} D_{B}=\frac{1}{8}{\left({\hat{x}}_{k|k}^{R1}-{\hat{x}}_{k|k}^{R2}\right)}^{T}P^{-1}\left({\hat{x}}_{k|k}^{R1}-{\hat{x}}_{k|k}^{R2}\right)+\frac{1}{2}ln\left(\frac{\text{det}P}{\sqrt{\text{det}P_{k|k}^{R1}\cdot \text{det}P_{k|k}^{R2}}}\right)\\ P=\frac{P_{k|k}^{R1}+P_{k|k}^{R2}}{2} \end{array}$$

Another critical issue of the algorithm is the object score update by using the introduction of some modifications when the information comes from another robot. Table 2 presents the scoring mechanism under different possible situations.

The implementation of all the previous concepts in the new algorithm brings about as the main advantage, in comparison with previous approaches in the literature [25,41], the capability for dealing with centralized or decentralized communication structures with no changes required (apart from communication issues, such as broadcast or point-to-point communication schemes).

Algorithm 4 describes the complete single robot object tracking process previously discussed in a systematic manner.

## 4. Results

A selection of tests were carried out in order to evaluate the performance of the algorithms within the modular structure proposed in this work. The purpose of these experiments was to identify and track the position and size of some dynamic objects. Tests in both simulated and real environments were carried out.

Section 4.1 shows the results obtained by using computer simulation in a series of CIs generated by using the Webots platform [42]. This simulation system takes into account not only sensor errors, but also typical errors in position and attitude estimates according to state-of-the-art navigation techniques.

**Figure 9.**
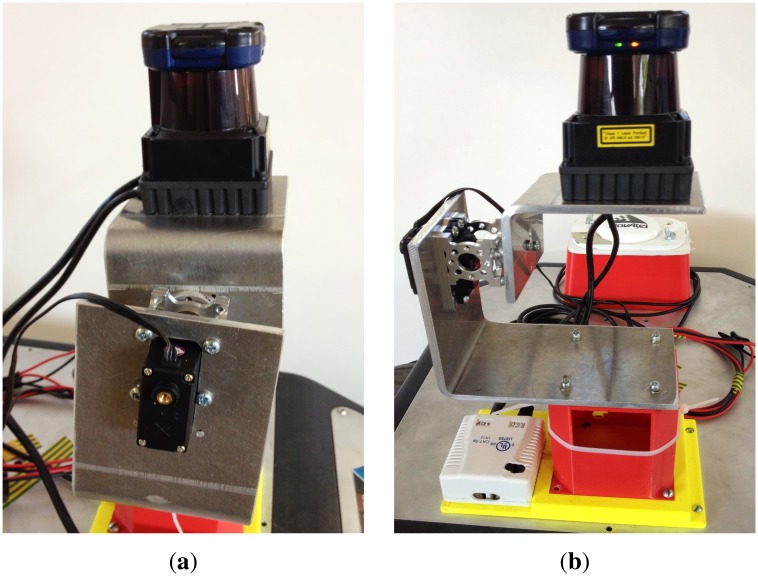
Hokuyo laser and tilt unit mounted on top of the Summit–XL robot. (**a**) Side view; (**b**) front view.

On the other hand, Section 4.2 describes some tests carried out by using a Summit–XL robotic platform equipped with a Hokuyo UTM-30LX range finder mounted on a tilt unit in a real scenario. This combination can be appreciated in Figure 9. The main drawback of this combination is that it has a lower frequency than a real 3D LiDAR, such as, for example, the Velodyne HDL-64E. This results in a lower velocity range for the detected objects. However, for the purposes of algorithm validation, the used sensor turned out to be fast enough.

Using these testing platforms, different detection and tracking exercises have been carried out to prove the performance of the algorithms. For simplicity, only some selected examples will be shown here to specifically highlight their robustness under occlusions and for long surveillance distances, two of the main problems encountered during DATMO in CIs. For better comprehension, the selected tests describe increasingly complex situations and trajectories, from very simple linear trajectories to more realistic movements and scenes. To critically evaluate the feasibility of the modular integration between the detection and tracking algorithms, examples involving different situations are shown.

### 4.1. Simulations

**Figure 10.**
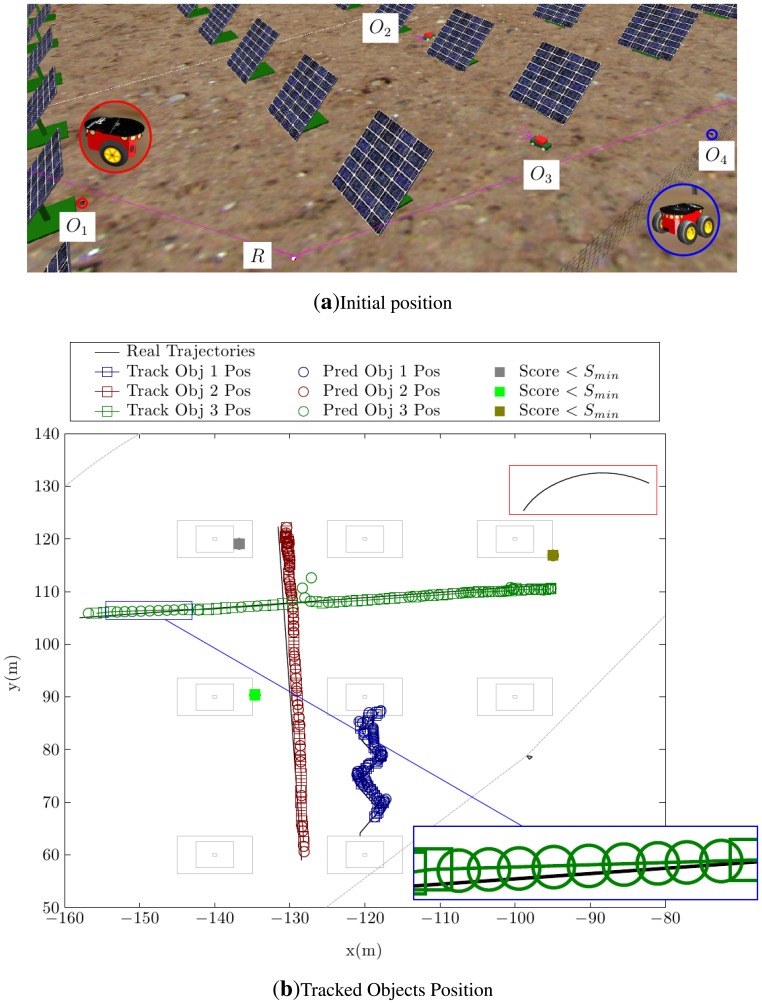
Simulation in an environment with obstacles. (**a**) The initial position of the elements and the surrounding environment is shown. (**b**) The tracked positions of the dynamic objects are show

A single robot must be able to detect and track multiple dynamic objects in an environment with obstacles and to follow their trajectories correctly. The first test is carried out in a simulated solar plant. The initial position of the elements in this test is shown in Figure 10a. For this test example, the range of the camera was set to 80 m.

Figure 10b shows the tracked positions for the detected objects. In this drawing, the ground layout of the solar panels is plotted for clarity, although the detecting robot did not have this information during the test. The detected trajectories plotted in this figure demonstrate that three out of four objects in the scene are perfectly detected, along with a couple of miss-detections located in the base of some of the solar panels. The fourth object is a simulated Pioneer-3AT robot (0.5 × 0.5 × 0.3 m) located about 50–60 m from the detector robot. This object, following the trajectory marked with a red rectangle in the figure, has not been detected. This failure in the detection is not due to the performance of the algorithm, but stems from the small size of the object and its long distance (*i.e.*, more than 60 m) from the detecting robot. Under these conditions, only very few laser beams impact the object, making it impossible to discern between its movement and noise-induced changes in the laser measurements.

As shown in Figure 10b, the tracking algorithm can deal with occlusions. The trajectory of Object 3 is a perfect example of how this algorithm deals with these situations. The zoomed area shows an occlusion and how the tracking algorithm predicts the position of the object in that zone until the detection algorithm finds it again.

**Figure 11.**
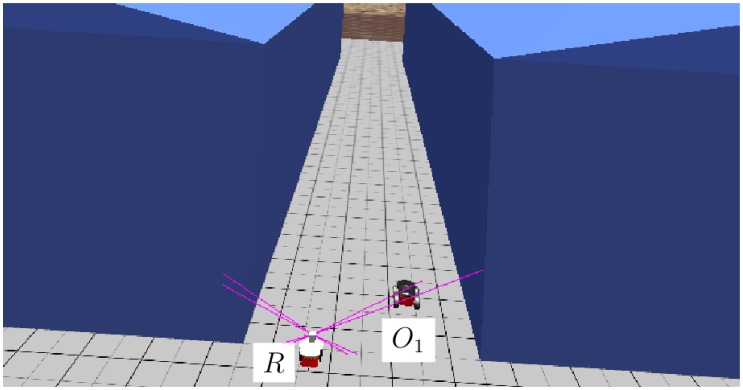
Initial position of the elements before the test of an object moving between walls.

Not just obstacles can be used to conceal possible intruders. Buildings can also be good hiding places for intruders moving along the walls to avoid detection. Figure 11 shows the initial position of the elements in a simulation in which a robot tracks a dynamic object between two walls. This situation could also represent a robot making a surveillance patrol between shelves in a storage area or between rows of containers in a harbor docking area.

**Figure 12.**
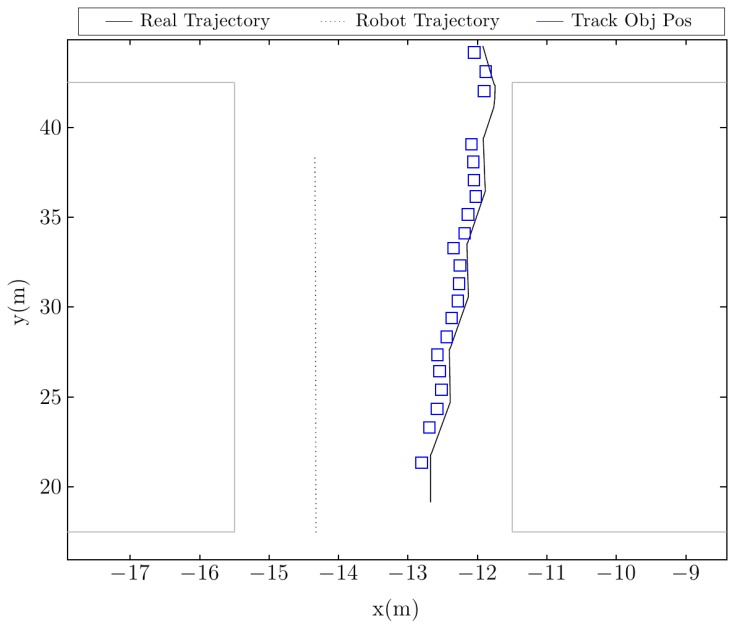
Tracked position of the moving object, as well as the trajectory of the robot.

Figure 12 shows the tracked trajectory of the moving object. Although the frequency of the detection is smaller than in other cases, the robot still manages to track the intruder. The main problem when trying to detect dynamic objects between or close to walls is that collision points in walls tend to have very different heights for the same cell in the ground reconstruction. These wall collisions may sometimes mask a dynamic object that moves close to them. To solve this problem, an efficient gradient calculation was included in the algorithms, allowing one, in this manner, to efficiently discern the cells that belong to the vertical walls.

**Figure 13.**
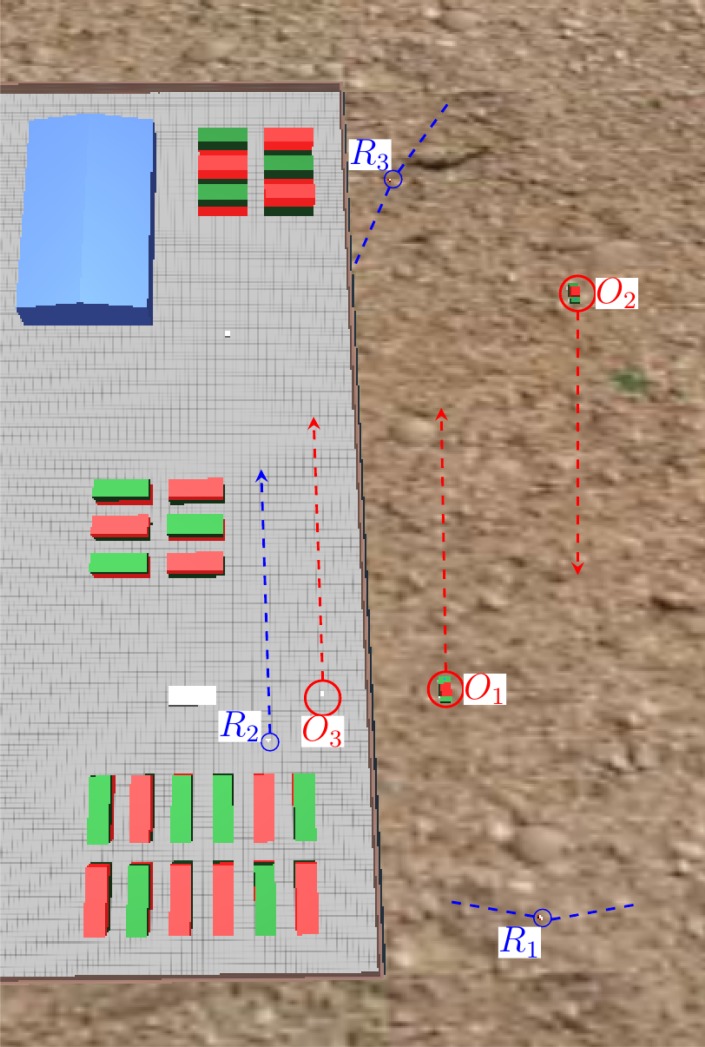
Detected object positions by different robots in a centralized structure. The object positions detected by each robot are marked with a different color.

The final purpose of a robot surveillance system is to convey all the information to a central station, where it must be presented to a human operator in a comprehensive way. To check this functionality, a successful simulation in which multiple robots transmit their DATMO information to a central station is required. To prove this capability of the algorithms, a test is carried out with three robots and three moving objects. Figure 13 shows the initial location of the elements in the test. Two robots are performing surveillance outside a perimeter wall, whereas the third one is patrolling the internal side of the same wall.

The central control station is simulated as a robot with only its tracking module active. The unique inputs to this control station are the moving object lists generated by the robots performing surveillance.

**Figure 14.**
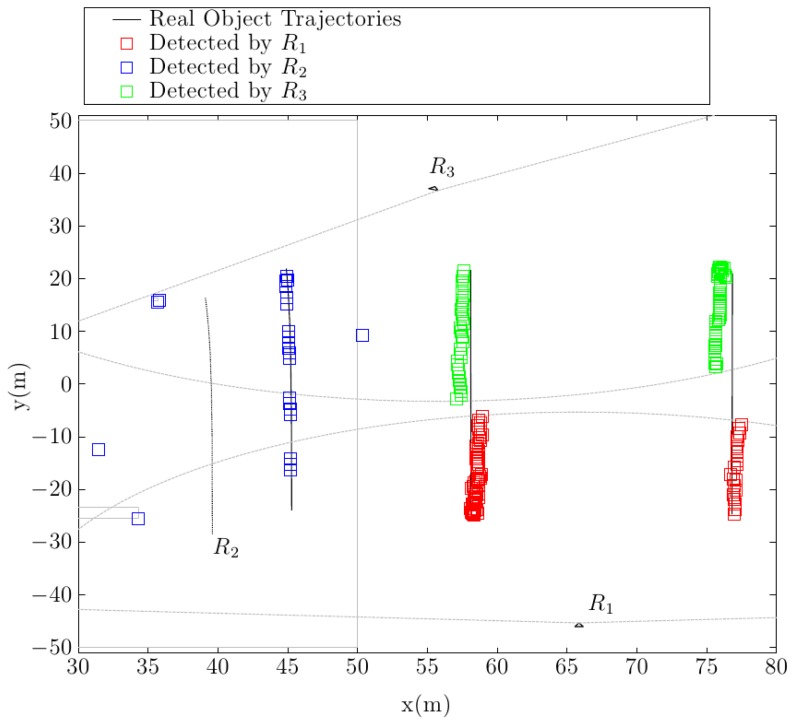
Detected object positions by each robot in the central station simulation.

Figure 14 shows the object positions detected by each surveillance robot. The contour lines of the static elements in the simulation, as well as the range of detection limits for the static robots are marked in the same plot for visualization purposes. It is important to notice that the robots have no information about static objects in the environment. All three robots detect the dynamic objects that are moving in their respective detection ranges. The dynamic robot, R2, is the only one that produces some detection errors. These errors appear mainly at obstacle corners, where the gradient conditions do not perform well in ruling those obstacles out. However, the tracking algorithms eliminate these miss-detections.

**Figure 15.**
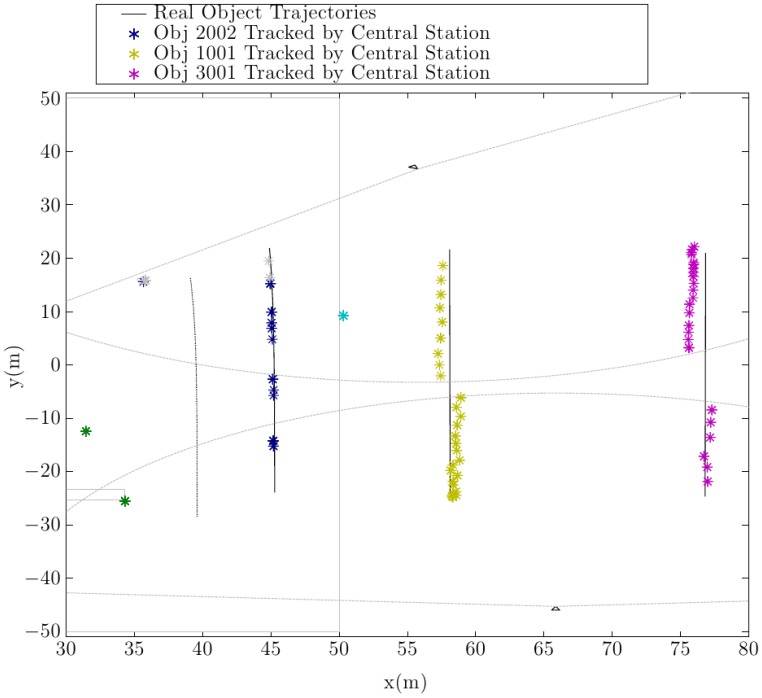
Tracked object positions by the central control station.

In this test, the central station continuously receives the dynamic object list generated by each robot. Figure 15 shows the object positions tracked by the central control station. It is apparent that the multirobot tracking algorithm efficiently combines the object lists and efficiently tracks different mobile objects, even when they move in areas covered by the the detection ranges of different robots. It is important to notice that for all detected robots, object identifications numbers (IDs) remain unaltered through the whole simulation. This ID corresponds to the ID given to the object by the first detecting robot.

### 4.2. Field Tests

The 3D range data necessary for the ground robot tests were obtained by combining a 2D LiDAR sensor with a vertical tilt movement produced by a servo, as presented previously. This system provides point clouds of 42,500 points. Of course, this arrangement has a lower resolution than that steaming from real 3D range devices, such as the HDL-32E device from Velodyne, which has an output of about 700,000 points. It must be stressed that the proposed algorithms could also handle such point clouds without any problem and that, in this case, their performance will be greatly increased. Nevertheless, for validation purposes, the used sensor data turned out to be good enough to demonstrate the performance of the algorithms.

**Figure 16.**
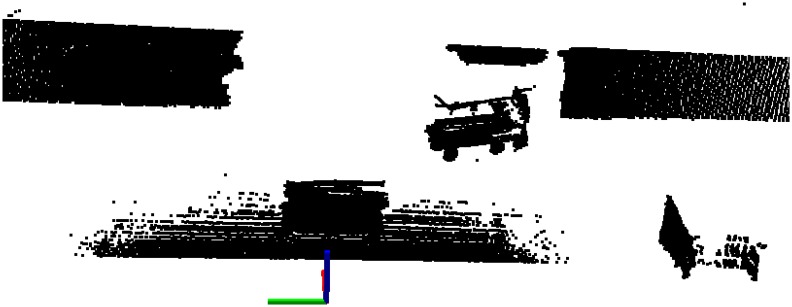
Point cloud of the initial situation of the test.

**Figure 17.**
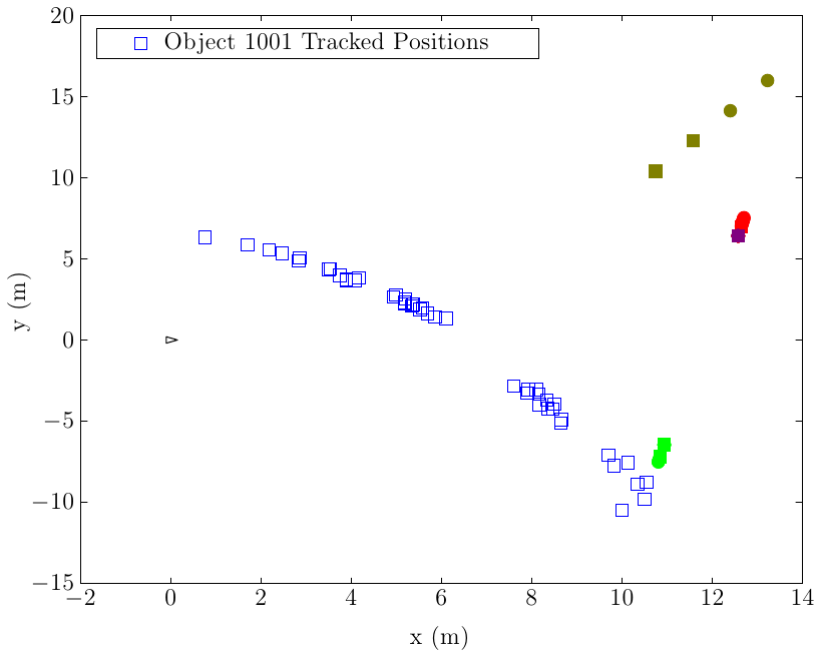
Tracked position of the moving objects.

The first test was performed by moving a big object in front of the robot. In this case, a car-sized object moved in front of the robot from right to left, whereby it was occluded at some point by a static obstacle. A point cloud taken at the beginning of the test can be seen in Figure 16. The evaluation results of this test can be seen in Figure 17. An occlusion can be clearly observed between the points, (7, 4) and (6, 2), when the object was passing behind the obstacle. Outside this area, the object was detected correctly along the whole trajectory. Some small gaps can also be observed in the trajectory that do not correspond to occlusions. These discontinuities are produced, because the determined center of big objects does not always correspond to the object's real center. These little inconsistencies originate, because the center of the object is estimated in different positions at different viewing angles.

**Figure 18.**
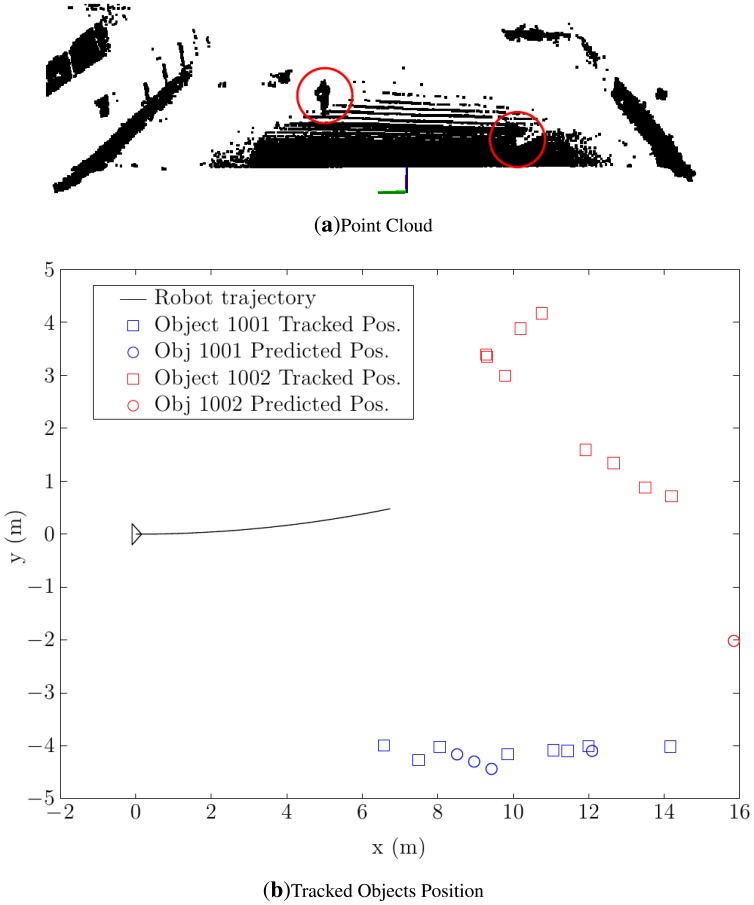
Real environment with obstacles. (**a**) The initial position of the elements and the surrounding environment is shown. (**b**) The tracked position of the moving objects can be seen.

Of course, when the robot is moving, its ability to detect and track moving objects must remain intact, and therefore, the dynamic detection algorithm must be also tested with real data. To illustrate its performance, the dynamic detection algorithm was tested in an exterior environment with two objects (i.e., persons in this test) moving in the detection range of the robot. A point cloud of the position of the elements at a half period test can be seen in Figure 18a.

The output of the tracking module for this test can be seen in Figure 18b. The two object trajectories are clearly visible, and the tracking algorithm identifies the objects correctly, even after object 1002 changes its direction at some moment during the test.

This latter test is a good example of how a robot patrolling the perimeter of a CI can effectively detect and track different objects. Even if the detection frequencies are lower than in the static case, all the objects are properly being detected without interruptions.

## 5. Conclusions

In this work, we have developed a series of algorithms to detect and track multiple mobile objects in large surveillance areas using a team of multiple robots. It has been proven that a good approach for DATMO is to use a modular structure for the calculations in which detection and tracking algorithms are run in a separate, but communicative way. This would permit us to use different detection algorithms and a unique tracking algorithm, provided that the data are supplied in a convenient form. The validity of the approach has been proven by combining two detection and two tracking algorithms in multiple tests.

Two different methods, intended for either a static or a dynamic robot, have been developed to detect dynamic objects. These algorithms, supplied with range images of the environment (2.5D data), are able to handle large surveillance environments and have proven to be efficient for detecting multiple dynamic objects in the presence of occlusions. For tracking purposes, two identification algorithms have been developed. The first one relies on well-established techniques aiming at identifying the detected dynamic objects within a local list in the robot. For this purpose, we combine the information from different sensors using a time-dependent Kalman filter to predict the next state of each object. Position, size and color are used to efficiently identify and track the objects by a nearest neighbor search with the Mahalanobis distance. This flexible algorithm could be expanded to incorporate other parameters, such as temperature, to track the objects. The second tracking algorithm combines lists of dynamics objects from different robots. Since the objects in these lists have different detection times and their parameters different covariances, the Mahalanobis distance cannot be applied. To overcome this limitation, the pairing of object lists is carried out by applying the Bhattacharyya distance to the nearest neighbor search. This approach has the advantage of enabling dissimilar robots to combine their knowledge about the moving objects (e.g., possible threats of the environment). This combination enables a better characterization of the dynamic objects. Another advantage of the modular combination of the present approach is that it could be used not only with a centralized, but also with a decentralized, surveillance architecture. Since the developed sharing procedure is fully scalable to a variable number of robots, it could be applied without incorporating additional equipment in the control center. This flexibility of the method would permit its use on an *à la carte* basis, whereby a variable number of robots could be used in a given environment by just making small adjustments to the software.

## Figures and Tables

**Table 1.** Scoring table for single robot tracking.

**Action**	**Scoring**
New object	1
Paired object	$+1-\frac{D_{M}}{D_{M|\text{max}}}$
Object not paired	−0.5

**Table 2.** Scoring criteria for multirobot tracking.

**Action**	**Scoring**
New object	Object score in previous list
Paired object	$+0.5\left(1-\frac{D_{B}}{D_{B|\text{max}}}\right)$
Object not paired	0
